# TREM2 in Neurodegenerative Diseases: Mechanisms and Therapeutic Potential

**DOI:** 10.3390/cells14171387

**Published:** 2025-09-05

**Authors:** Ling Li, Xiaoxiao Zheng, Hongyue Ma, Mingxia Zhu, Xiuli Li, Xiaodan Sun, Xinhong Feng

**Affiliations:** 1 Department of Neurology, Beijing Tsinghua Changgung Hospital, School of Clinical Medicine, Tsinghua Medicine, Tsinghua University, Beijing 102218, China; liling24@mails.tsinghua.edu.cn (L.L.); zxxs02476@btch.edu.cn (X.Z.); mhys02007@btch.edu.cn (H.M.); 15730664785@163.com (M.Z.); lxla00834@btch.edu.cn (X.L.); 2 Key Laboratory of New Ceramics and Fine Processing, School of Materials Science and Engineering, Tsinghua University, Beijing 100084, China; sunxiaodan@tsinghua.edu.cn; 3 Key Laboratory of Advanced Materials of Ministry of Education of China, School of Materials Science and Engineering, Tsinghua University, Beijing 100084, China

**Keywords:** TREM2, neurodegenerative disease, microglia, therapy

## Abstract

Neurodegenerative diseases, including Alzheimer’s disease (AD), Parkinson’s disease (PD), and amyotrophic lateral sclerosis (ALS), represent significant global health challenges, affecting millions and straining healthcare systems. These disorders involve progressive neuronal loss and cognitive decline, with incompletely elucidated underlying mechanisms. Chronic neuroinflammation is increasingly recognized as a critical contributor to disease progression. The brain’s resident immune cells, microglia, are central to this inflammatory response. When overactivated, microglia and other immune cells, such as peripheral macrophages, can exacerbate inflammation and accelerate disease development. Triggering Receptor Expressed on Myeloid Cells 2 (TREM2) is a transmembrane receptor of the immunoglobulin superfamily that demonstrates high expression on microglia in the central nervous system. TREM2 serves a vital role in regulating phagocytosis, synaptic pruning, and energy metabolism. This review examines the functions of TREM2 in neurodegenerative diseases and its potential as a therapeutic target, aiming to inform future treatment strategies.

## 1. Introduction

Neurodegenerative diseases, including Alzheimer’s disease (AD), Parkinson’s disease (PD), and amyotrophic lateral sclerosis (ALS), constitute a critical global public health challenge. These disorders are characterized by progressive neuronal loss and cognitive or motor decline—sometimes simultaneously, as in Parkinson’s disease dementia (PDD)—leading to severe disability, substantial caregiver burdens, and ever-increasing socioeconomic and healthcare costs [1]. AD alone accounts for 60–70% of global dementia cases, with projections indicating there will be over 150 million affected individuals by 2050. Despite extensive research, the pathogenic mechanisms driving neurodegeneration remain incompletely understood. While hallmark pathologies such as amyloid plaques, neurofibrillary tangles, and α-synuclein aggregates have been identified, multiple hypotheses exist, including the amyloid cascade, neuroinflammation, vascular and infection-related mechanisms, and the recently proposed multi-sensor theory [2]. Current therapies primarily address symptoms without halting disease progression, highlighting the urgent need for novel therapeutic targets [3].

Emerging evidence indicates chronic neuroinflammation is a fundamental driver of neurodegenerative processes [4]. Microglia, the brain’s resident immune cells, serve dual roles in maintaining homeostasis by phagocytizing pathological debris, regulating synaptic plasticity, and modulating inflammatory responses [5]. However, under pathological conditions, sustained microglial activation induces excessive release of pro-inflammatory cytokines (e.g., Interleukin-1 beta (IL-1β) and TNF-α), reactive oxygen species, and complement system proteins, establishing a self-perpetuating inflammatory cascade. This dysregulation is further amplified by peripheral macrophage infiltration and astrocytic reactivity, collectively creating a neurotoxic microenvironment that accelerates synaptic loss and neuronal death. Such immune–neural network imbalances not only propagate amyloid-β (Aβ) and tau pathologies but also intersect with metabolic dysfunction and impaired autophagy, forming a multifaceted pathogenic framework [6,7].

Within this context, Triggering Receptor Expressed on Myeloid Cells 2 (TREM2), a key regulator of microglial function, has emerged as a significant focus [8]. As a member of the immunoglobulin superfamily, TREM2 activates downstream DNAX-activating protein 12 (DAP12)/Spleen Tyrosine Kinase (SYK) signaling upon binding ligands, such as lipids, apoptotic cells, and Aβ aggregates, thereby modulating phagocytosis, inflammatory responses, and metabolic adaptation [9]. Genome-wide association studies (GWASs) have identified loss-of-function *TREM2* variants (e.g., *R47H*) as significant genetic risk factors for AD, frontotemporal dementia, and Nasu–Hakola disease [10]. Additionally, elevated cerebrospinal fluid levels of soluble TREM2 (sTREM2) correlate with neurodegenerative biomarkers (e.g., tau), suggesting its potential as a dynamic indicator of disease activity [11]. Preclinical studies have demonstrated that TREM2 agonists enhance microglial clearance of pathological proteins and promote metabolic resilience, reducing amyloid burden and cognitive deficits [12]. This review comprehensively examines the multifaceted roles of TREM2 in neurodegenerative diseases, evaluates its therapeutic potential, and provides direction for developing innovative immunometabolic interventions.

## 2. Materials and Methods

We conducted a systematic literature search of the PubMed and Web of Science databases to identify studies addressing TREM2 in neurodegenerative diseases (search period: January 2000–May 2025). The following keywords were used in different combinations: “TREM2”, “triggering receptor expressed on myeloid cells 2”, “neurodegenerative diseases”, “Alzheimer’s disease”, “Parkinson’s disease”, “frontotemporal dementia”, and “ALS”. Boolean operators (AND and OR) and database-specific filters were applied.

### 2.1. Inclusion Criteria

Peer-reviewed original research or review articles;

Articles written in English;

Studies focusing on TREM2 in the context of neurodegenerative diseases (mechanisms, pathology, or therapeutic potential).

### 2.2. Exclusion Criteria

Duplicates across databases;

Conference abstracts, editorials, book chapters, and non-peer-reviewed articles;

Articles lacking experimental or mechanistic insights (e.g., commentary-only articles).

### 2.3. Screening Process

The literature search yielded 3384 articles (PubMed: 1402; Web of Science: 1982). After duplicate removal, 1542 articles remained. Title and abstract screening excluded 1026 articles, leaving 516 for full-text review. Following comprehensive evaluation, 170 articles met the inclusion criteria and were incorporated into this review (128 original research studies and 42 review articles).

## 3. Structure of TREM2

**Figure 1:**
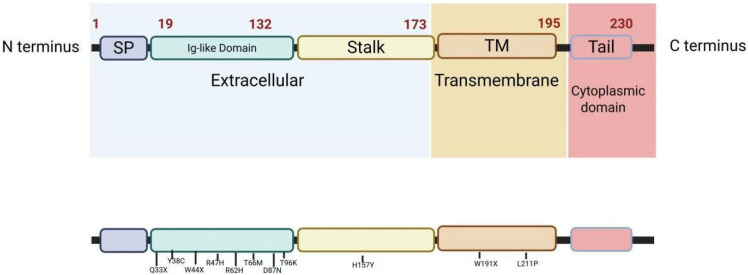
Structural architecture and pathogenic mutations of the TREM2 protein. This schematic illustrates the domain organization and disease-linked mutations of TREM2, a key receptor in microglial function and neurodegenerative disease pathology. TM = transmembrane; SP = signal peptide; and Ig = immunoglobulin-like. The images were created using licensed Biorender online software (https://app.biorender.com/).

TREM2 is a 26 kDa transmembrane immunoregulatory receptor encoded by the *TREM2* gene, located on human chromosome 6p21.1 and mouse chromosome 17C3. The TREM2 protein comprises five exons, encoding a full-length protein consisting of 230 amino acids [13,14]. TREM2 functions as a single-pass transmembrane receptor protein within the immunoglobulin superfamily. Its structure includes an extracellular immunoglobulin-like V-type domain, a stalk region, a helical transmembrane domain, and a short cytoplasmic tail lacking signaling and trafficking motifs [15,16]. Figure 1 illustrates the structure and pathogenic mutations of TREM2.

The extracellular domain of TREM2 consists of two primary structural components: an immunoglobulin (Ig)-like ligand-binding module composed of nine antiparallel β-strands and a proximal stalk region [17]. This ligand-binding domain contains essential post-translational modification sites, including N-linked glycosylation, disulfide bonds, and phosphorylation motifs. The transmembrane segment incorporates conserved lysine residues that facilitate interaction with adaptor proteins DAP12 and DAP10 through electrostatic coupling [18]. These positively charged residues establish salt bridges with aspartic acid residues in the transmembrane helices of DAP12/DAP10, forming a stable heterodimeric signaling complex essential for signal transduction [16]. Although TREM2’s short intracellular C-terminal domain lacks inherent signaling capacity, the transmembrane lysine residues are essential for maintaining the DAP12/DAP10 interaction [19]. Biochemical analyses have demonstrated that TREM2 undergoes constitutive membrane shedding with a brief half-life (<1 h), producing soluble TREM2 (sTREM2) fragments under normal conditions [20]. Within the central nervous system (CNS), TREM2 exhibits widespread neuroanatomical distribution, with notable expression in the basal ganglia, corpus callosum, and spinal cord [21,22]. Cellular expression studies have identified myeloid lineage cells, particularly immature dendritic cells and tissue-resident macrophages, as primary expressors [23].

The Ig-like domain facilitates diverse ligand interactions through three primary mechanisms: (1) pattern recognition of bacterial components (e.g., lipopolysaccharide); (2) phospholipid sensing via phosphatidylserine binding; and (3) molecular chaperone interactions with apolipoproteins, including Apolipoprotein E (APOE) [24,25]. In the pathogenesis of AD, the extracellular domain specifically recognizes β-amyloid aggregates, initiating DAP12/DAP10-dependent signal transduction cascades upon ligand engagement [26].

## 4. TREM2 Signaling Pathways

**Figure 2:**
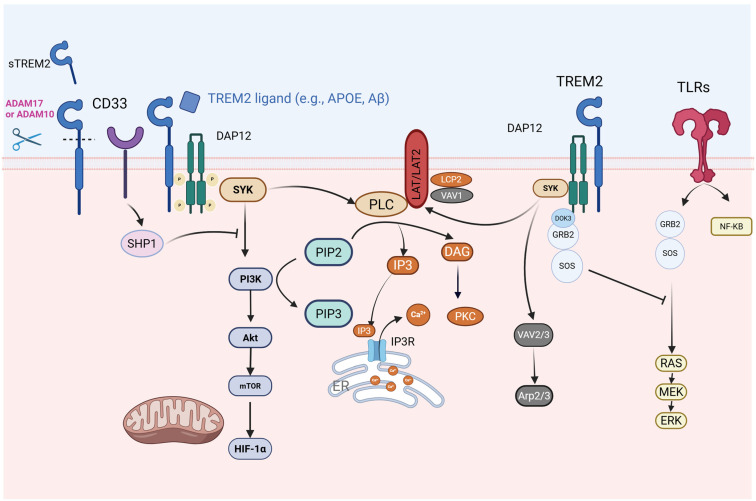
TREM2-DAP12 signaling pathway and its downstream regulatory network. This diagram illustrates the immune-related signaling cascade triggered by TREM2 upon binding to its ligands. The pathway involves the following key mechanisms. The images were created using licensed Biorender online software (https://app.biorender.com/).

DAP12 contains an immunoreceptor tyrosine-based activation motif (ITAM). When TREM2 is activated, the tyrosine residues within the ITAM of DAP12 undergo phosphorylation, triggering the recruitment and activation of tyrosine protein kinase SYK. SYK subsequently activates downstream signaling molecules, including Vav guanine nucleotide exchange factors, non-receptor tyrosine kinase Pyk2, phosphatidylinositol 3-kinase (PI3K), phospholipase Cγ (PLCγ), and membrane-bound adaptors such as LAT1/2. PI3K, in an action facilitated by DAP10, localizes to the membrane, where it catalyzes the conversion of phosphatidylinositol-4,5-bisphosphate (PIP2) into phosphatidylinositol-3,4,5-trisphosphate (PIP3). This PI3K-mediated conversion activates PLCγ, which subsequently converts PIP2 into inositol trisphosphate (IP3) and diacylglycerol (DAG). The TREM2–DAP12–DAP10 signaling cascade initiates a series of protein and lipid phosphorylation events, resulting in Ca^2+^ mobilization, integrin activation, cytoskeletal rearrangement, activation of the mechanistic target of rapamycin (mTOR) and mitogen-activated protein kinase (MAPK) signaling, and enhanced energy metabolism [18,27]. DAP10’s intracellular domain contains a tyrosine–isoleucine–asparagine–methionine motif (YINM), which enables the recruitment of p85, a regulatory subunit of PI3K, promoting PIP2-to-PIP3 conversion. Furthermore, activated TREM2 partially phosphorylates DAP12’s ITAM region, creating binding sites for the Src homology 2 (SH2) domains of SYK and Zeta-chain-associated protein kinase 70 (ZAP70) kinases. Simultaneously, inositol phosphatase-1 (SHIP1) competes with SYK for ITAM binding, thereby reducing DAP12-SYK and DAP10-PI3K interactions, ultimately suppressing downstream immune responses [28,29]. Figure 2 illustrates the TREM2-DAP12 pathway and the downstream regulatory network.

### 4.1. TREM2-Toll-like Receptor 4 (TLR4)/MAPK Axis in Neuroinflammatory Regulation

Recent evidence has demonstrated the critical role of TREM2 dysregulation in the pathogenesis of neuroinflammation. Studies using APP/PS1 transgenic mice and BV2 microglial models have revealed that TREM2 deficiency aggravates AD pathology through TLR4-dependent MAPK pathway activation, resulting in accelerated Aβ deposition, enhanced gliosis around amyloid plaques, and substantial neuronal/synaptic protein loss alongside cognitive decline. At the molecular level, TREM2 downregulation initiates a neuroinflammatory cascade by promoting TLR4/MAPK-mediated production of pro-inflammatory cytokines in both cellular and organismal systems [30]. The neuroinflammatory responses triggered by TREM2 reduction may contribute to the pathological mechanisms of AD. Significantly, TREM2 overexpression studies in intracerebral hemorrhage models have indicated therapeutic potential: elevated TREM2 expression provided neuroprotection through multiple mechanisms, namely, reducing cerebral edema, inhibiting neuroinflammatory responses via TLR4/NF-κB/MAPK pathway suppression, and reducing apoptotic processes [31]. This variation may result from differential activation of TLR4 by damage-associated molecular patterns (DAMPs) in the pathological microenvironment.

### 4.2. TREM2 and Calcium-Modulated Signaling Pathways

TREM2 activates downstream signaling pathways, including calcium signaling, through its association with DAP12. TREM2 transmits signals via its co-receptor DAP12, activating downstream signaling molecules, including PLCγ2. PLCγ2 activation leads to PIP2 hydrolysis, generating IP3 and DAG. IP3 binds to IP3 receptors on the endoplasmic reticulum (ER), triggering the release of stored calcium ions into the cytoplasm, thus increasing intracellular calcium ion concentrations. The elevated calcium ions can facilitate the assembly and activation of the NOD-like receptor family pyrin domain containing 3 (NLRP3) inflammasome, a multi-protein complex involved in IL-1β maturation and secretion [32]. These alterations are essential for microglial function [25]. Obst et al. demonstrated that *PLCγ2* deficiency results in cell adhesion and migration defects in human-induced Pluripotent Stem Cell (iPSC)-derived macrophages, likely through integrin-dependent mechanisms. These findings suggest PLCγ2 is crucial for various cellular responses, supporting the hypothesis that PLCγ2 modulation could serve as a potential therapeutic strategy for microglial function in AD [33]. Jairaman et al. discovered that iPSC-derived microglia lacking *TREM2* exhibit hyperactive Ca^2+^ signaling in response to adenosine agonists, such as ADP, affecting microglial injury responses. This ADP hypersensitivity results from increased P2Y12 and P2Y13 receptor expression, leading to increased release of stored Ca^2+^ from the ER, triggering sustained Ca^2+^ influx through Orai channels and altering *TREM2* knockout (KO) microglia motility. P2Y12 receptor antagonists can restore the chemotaxis defect in *TREM2* KO microglia by reducing cytosolic Ca^2+^ [34]. These findings indicate that the absence of TREM2 leads to defective Ca^2+^ responses to adenosine signals in microglia, suggesting there is an optimal Ca^2+^ signaling window for microglial motility [34].

### 4.3. TREM2 and SHIP1-Related Signaling Pathways

Upon activation, TREM2 signals through the adaptor molecule DAP12, which contains an ITAM. When TREM2 is activated, the ITAM of DAP12 recruits and activates the tyrosine protein kinase SYK. SHIP1 regulates microglial function and activation by modulating different but converging signaling pathways. SHIP1 inhibits TREM2 signaling by binding to the ITAM of DAP12 in osteoclasts, preventing the p85 regulatory subunit of PI3K from binding to the ITAM of DAP12. This indicates that SHIP1 inhibits PI3K-mediated signaling events downstream of TREM2/DAP12 [29,35]. Research using human microglia-like HMC3 cells expressing wild-type *TREM2*, the *R47H* variant, and *TREM2*-deficient cells demonstrated that functional TREM2 is essential for Aβ phagocytosis, lysosomal capacity, and mitochondrial activity. SHIP1, which regulates TREM2 signaling in other cells, functions as a negative regulator of these TREM2-mediated processes. Moreover, pharmacological inhibition of SHIP1 and its isoform Src Homology 2-containing Inositol Phosphatase 2 (SHIP2) enhances Aβ phagocytosis and lysosomal capacity, independently of TREM2 or SHIP1 expression, suggesting SHIP2’s regulatory role in these functions [36].

### 4.4. Crosstalk Between TREM2 and CD33

Research has demonstrated that the microglial receptors CD33 and TREM2 are associated with the risk of developing AD. In 5xFAD mouse models, *CD33* KO mitigated Aβ pathology and enhanced cognitive function, though these effects diminished when *TREM2* was also knocked out. *TREM2* knockout exacerbates Aβ pathology and neurodegeneration while reducing Iba1+ cell numbers, effects that persist despite additional *CD33* KO. RNA-seq analysis revealed upregulation of genes related to phagocytosis and signaling, including Interleukin-6 (IL-6), IL-8, and acute-phase response in 5xFAD; *CD33*^−/−^ mice, while these genes were downregulated in 5xFAD; *TREM2*^−/−^ mice, indicating TREM2’s downstream position relative to CD33. The CD33-TREM2 crosstalk encompasses regulation of the IL-1β/IL-1RN axis and the “receptor activity chemokine” gene set. These insights contribute to the development of AD therapies targeting these receptors [37]. Studies have indicated that CD33 inhibits Syk activation through SHIP1 interaction, thereby suppressing PI3K activation, autophagy, and microglial metabolic homeostasis [38,39]. Additionally, CD33 and TREM2 exhibit complex interactions that may influence microglial Aβ uptake and degradation.

TREM2 operates within a complex network. Its interactions with CD33- and SYK-dependent signaling potentially determine microglial functional states. These relationships are crucial for developing combinatorial therapies but remain oversimplified in the literature. Further investigation of the intricate interactions between TREM2-, CD33-, and SYK-dependent signaling is necessary.

## 5. Soluble TREM2 (sTREM2)

Soluble TREM2 (sTREM2), generated through proteolytic cleavage of the full-length protein by metalloproteinase 10 (ADAM10) and ADAM17, is a biologically active molecule capable of ligand binding, microglial activation, and immune response modulation in the AD continuum [40]. In clinical settings, cerebrospinal fluid (CSF) sTREM2 functions as a dynamic biomarker indicating microglial activation intensity, with elevated levels in AD patients correlating strongly with neurodegeneration markers (T-tau and P-tau181) [11]. Mendelian randomization analyses have established causal associations between genetically elevated CSF sTREM2 and the risk of developing multiple sclerosis [41], while case–control studies have revealed its diagnostic potential in ALS through elevated serum/CSF levels [42]. These observations establish sTREM2 as a pan-neuroinflammatory biomarker valuable for disease stratification and therapeutic monitoring. Mechanistically, Zhong et al. demonstrated sTREM2’s therapeutic efficacy in AD models, where it mitigates amyloid pathology through microglial reprogramming, enhancing plaque surveillance and Aβ clearance, subsequently improving synaptic plasticity and cognitive function. Notably, microglial depletion nullifies these benefits, confirming the necessity of intact microglial responses for sTREM2-mediated functional restoration [43]. Belsare et al. demonstrated that sTREM2 specifically interacts with fibrillar Aβ40 and Aβ42 with micromolar affinity rather than monomeric Aβ, selectively inhibiting secondary nucleation in fibrillization. Additionally, sTREM2 enhances cellular uptake of Aβ fibrils, a function compromised by the AD-associated *R47H* mutation, indicating a structural basis for its activity [44]. The dual functionality of sTREM2 may relate to the ADAM10/17 cleavage site and disease progression stages [40,45]. While there is an increasing amount of evidence supporting sTREM2’s critical role in neurological disorders, further research is needed to fully understand its mechanisms in modulating microglial function across different pathological contexts and disease stages as well as its interaction with membrane-bound TREM2 [46].

## 6. TREM2 Variants

Genetic variants in *TREM2* constitute established risk factors for multiple neurodegenerative diseases, including AD, PD, ALS, and frontotemporal dementia (FTD) (Table 1). The significance of TREM2 was initially revealed through the discovery of the fact that biallelic loss-of-function mutations cause Nasu–Hakola disease (NHD), a rare autosomal recessive disorder characterized by early-onset dementia and bone lesions [47,48,49]. Research on NHD-associated mutations has established that complete loss of TREM2 function results in severe microglial dysfunction, including impaired lysosomal activity [50].

The heterozygous *R47H* variant has emerged as a significant risk factor for several common neurodegenerative diseases. This variant substantially increases the risk of late-onset AD, associated with decreased microglial coverage of amyloid plaques, enhanced neuritic dystrophy, and compromised phagocytosis [51,52]. The *R47H* variant’s risk effect extends to FTD, PD, and ALS [53,54], demonstrating its broad impact on neuroimmune homeostasis.

**Table 1:** Summary of TREM2 genetic variants in neurodegenerative diseases.

Variant	Mutation Type	Associated Diseases	Domain/Location	Functional Impact	Clinical Significance	References
*R47H*	Missense	AD, ALS	Coding Exon 2	Reduces binding to APOE, Aβ, and lipoproteins; impairs SYK/PI3K signaling and phagocytosis.	Increases AD risk (OR ≈ 2–3); linked to diffuse amyloid plaques and axonal dystrophy.	[54,55]
*R62H*	Missense	AD, FTD	Coding Exon 2	Disrupts dimerization and ligand binding; reduces signaling efficiency.	Moderately increases AD risk (OR ≈ 1.5).	[56,57]
*T66M*	Missense	FTD/FTLDNHD	Coding Exon 2	Results in misfolding of the TREM2 protein and loss of function.	Results in early-onset dementia and bone cysts.	[58,59,60,61]
*Y38C*	Frameshift mutation	FTD/FTLDNHD	Coding Exon 2	Alters the flanking sequence of a cysteine for the Ig V-fold interchain disulfide bond; disrupts protein function.	Severe early-onset NHD with dementia and bone lesions.	[58,62,63]
*Q33X*	Nonsense	NHD, AD	Coding Exon 2	Produces truncated proteins, resulting in a complete loss of protein function.	Homozygous: NHD; heterozygous: increases AD risk.	[58,64]
*D87N*	Missense	AD	Coding Exon 2	Reduces APOE binding but enhances signaling in some contexts.	Increases AD risk in controversy.	[55]
*T96K*	Missense	AD, FTD	Coding Exon 2	-	A significant genetic risk factor for late-onset AD (LOAD) in the Tunisian population.	[65,66]
*H157Y*	Missense	AD	Coding Exon 3	Increases shedding of sTREM2. Reduces activation in response to phospholipid ligands and decreases phagocytosis.	Increases risk of AD.	[67,68]
*L211P*	Missense	FTD	Coding Exon 4	-	Increases risk of FTD, especially bvFTD.	[65]
*W44X*	Nonsense	NHD	Coding Exon 2	Produces non-functional truncated proteins that fail to activate microglia.	Strongly associated with NHD phenotype.	[47]
*W191X*	Nonsense	AD	Coding Exon 4	Stop-gain mutation.	Suggestive association with AD.	[69]

Analysis of these variants, particularly *R47H*, provides essential genetic evidence that even a partial reduction in TREM2 function—typically through impaired ligand binding—sufficiently disrupts key microglial signaling pathways. This disruption compromises critical functions, including lipid metabolism, neuronal damage response, and plaque compaction. The subsequent sections examine these downstream signaling pathways (e.g., SYK and PI3K-AKT), their mechanistic roles in disease, and the resulting therapeutic implications.

## 7. Impact of TREM2 on Microglial Function

### 7.1. Influence on Microglial Homeostasis and Activation

Ligand binding initiates downstream microglial signaling through transcriptional changes, transitioning these cells from a homeostatic state to a disease-associated microglia (DAM) state in neurodegenerative mouse models [70]. TREM2 plays a critical role in guiding microglia in the transition from a homeostatic state to an activated state in specific contexts, a transition that is essential for responding to neuroinflammation and demyelination. Single-cell analysis of DAM and TREM2 in transgenic Alzheimer’s disease (Tg-AD) models has demonstrated that the DAM program is activated in a two-step process. The initial activation occurs in a TREM2-independent manner, involving the downregulation of microglial checkpoints, followed by the activation of a TREM2-dependent program. This distinct microglial phenotype exhibits the potential to limit neurodegeneration, which may have significant implications for future treatments of AD and other neurodegenerative diseases [70]. TREM2 is fundamental for maintaining microglial metabolic adaptability during stress events, enabling microglia to develop into fully mature DAM and sustain their response to Aβ plaque-induced pathology [18]. Microglia adhering to Aβ plaques acquire a transcriptional signature known as “disease-associated microglia”, which primarily derives from the TREM2-DAP12 receptor complex. This complex transmits intracellular signals via the protein tyrosine kinase SYK. The human *TREM2 R47H* variant, associated with a high risk of AD development, cannot activate microglia via SYK. *SYK*-deficient microglia cannot envelop Aβ plaques, resulting in accelerated brain pathology and behavioral deficits. *SYK* deficiency impairs the PI3K-AKT-GSK-3β-mTOR pathway, leading to a loss of the anabolic support required for acquiring the DAM profile [71]. Single-nucleus transcriptomics studies have revealed that in 5xFAD mice, compared with wild-type (WT) mice, genes such as *Cst7*, *Csf1*, *Apoe*, *Trem2*, *Lpl*, *Lilrb4a*, *MHC-I (H2-d1)*, *MHC-II (Cd74)*, and various cathepsin genes are significantly upregulated. Conversely, homeostatic genes including *P2ry12*, *Selplg*, *Tmem119*, and *Cx3cr1* are downregulated. Furthermore, genes such as *Cst7*, *Csf1*, and *MHC-I (H2-K* and *b2m)* are significantly more expressed in 5xFAD microglia relative to *TREM2*^−/−^ 5xFAD microglia, highlighting the TREM2-dependent upregulation of these genes [72].

### 7.2. TREM2 and Microglial Metabolism

Metabolic flexibility or plasticity describes cells’ capacity to modify their nutrient metabolism in response to environmental changes. This adaptability is essential for maintaining cellular function. The polarization of immune cells following the activation of inflammatory responses requires the reprogramming of cellular metabolic pathways. This metabolic reprogramming is characterized by increased glycolytic flux, the accumulation of specific TCA cycle metabolites, reduced mitochondrial respiration, and enhanced transcriptional control by HIF-1α and mTOR. These alterations regulate microglial immune functions, including cytokine production and phagocytosis, contributing to neuroinflammation in the brain [73]. Research has demonstrated that TREM2 signaling regulates microglial energetics through the mTOR pathway in AD-like mouse models. Microglia from 5xFAD mice lacking TREM2 exhibit more autophagic vesicles compared to those from 5xFAD mice with functional TREM2. Similarly, microglia from AD patients with the *TREM2 R47H* display more autophagic vesicles than those with the common *TREM2* variant. Autophagy constitutes a crucial intracellular degradation pathway for cellular and energy homeostasis [74]. Metabolomic and RNA-seq analyses have revealed deficiencies in metabolites and enzymes involved in glycolysis, the TCA cycle, and the pentose phosphate pathway in *TREM2*^−/−^ bone-marrow-derived macrophages (BMDMs). These deficiencies can be counteracted in vitro by Dectin-1 and creatine. Dietary creatine modulates autophagy, enhances microglial clustering around plaques, and reduces plaque-associated neuritic dystrophy in *Trem2*-deficient mice with amyloid-β pathology. Thus, TREM2 facilitates microglial responses during AD by maintaining cellular energy and biosynthetic metabolism [27]. Treatment of BMDMs with a TREM2 agonist enhances mitochondrial potential [75]. During development, TREM2 expression in microglia maintains normal neuronal bioenergetics. Mice lacking TREM2 demonstrate impaired hippocampal neuronal bioenergetics during development, with developing neurons in the hippocampal CA1, but not CA3, subregion exhibiting impaired energy metabolism, reduced mitochondrial mass, and organelle ultrastructural abnormalities. These findings indicate that TREM2 regulates neuronal development by controlling metabolic adaptability in a region-specific manner [76]. When APP/PS1 mice, an AD model, consume a Western diet, microgliosis and increased TREM2 expression occur. This results in increased plaque burden, suggesting that altered microglial phagocytosis may explain how certain nutritional factors increase the risk of the progression of AD [77]. Long-term running exercises enhance cognitive function in the hippocampus in APP/PS1 mice, inhibit TREM2 shedding, and maintain TREM2 protein levels. Running exercises also increase FDG uptake and protein expression of GLUT5, TREM2, SPP1, and p-SYK in the hippocampus in APP/PS1 mice, correlating with improved brain glucose metabolism, microglial glucose metabolism, and hippocampal microglial morphological plasticity. These findings suggest microglia may be structural targets responsible for the benefits of running exercise in relation to AD. Therefore, enhancing TREM2-regulated microglial glucose metabolism and morphological plasticity could represent a novel AD treatment strategy [78].

TREM2 functions as a receptor for specific extracellular lipids [79]. Research has indicated that TREM2, as a lipid receptor, significantly influences adipose tissue macrophage responses in obese individuals. Single-cell genomic analysis has revealed that TREM2+ LAM (lipid-associated macrophage) cells represent the most expanded immune cell population in adipose tissue across multiple mouse models of obesity. The signature of TREM2 and associated genes involved in phagocytosis and lipid catabolism appears in macrophages across multiple organs in both mice and humans, suggesting this pathway represents a conserved response to irregular lipid composition, levels, and distribution. Furthermore, TREM2 signaling facilitates the formation of lipid-associated macrophages in crown structures within adipose tissue, preventing adipocyte hypertrophy and maintaining systemic lipid homeostasis under obese conditions. Complete removal of *Trem2* in mice inhibits the downstream LAM program, resulting in adipocyte hypertrophy, systemic hypercholesterolemia, fat accumulation, and glucose intolerance [80]. Recent evidence indicates that extracellular lipid–TREM2 interactions may regulate intracellular lipid metabolic programs at the transcriptional level, potentially maintaining lipid homeostasis by preventing pathological accumulation of phagocytosis-derived toxic metabolites. While *TREM2*-deficient microglia demonstrate intact myelin phagocytosis, they show impaired clearance of myelin-derived cholesterol, leading to cholesterol ester (CE) overload—a phenotype observed in *APOE*-deficient glia and replicated across *TREM2*^−/−^ murine macrophages, human iPSC-derived microglia, and foam cell models. Notably, pharmacological inhibition of Acyl-CoA Cholesterol Acyltransferase 1 (ACAT1) (blocking CE synthesis) or Liver X Receptor (LXR) agonism (enhancing cholesterol efflux) corrects this metabolic defect, establishing TREM2 as a key transcriptional regulator of cholesterol flux during chronic phagocytic challenges [26]. Studies using atherosclerosis models have demonstrated TREM2’s diverse roles: myeloid-specific *TREM2* deletion reduces oxLDL uptake in foam cells and diminishes plaque progression—even in established lesions—independent of systemic inflammation or lipidemia. Mechanistic analyses have shown that *TREM2* deficiency disrupts cholesterol efflux pathways (e.g., A member 1 (ABCA1) downregulation), compromising macrophage proliferative capacity and survival [81]. Conversely, therapeutic administration of TREM2 agonists increases plaque macrophage populations through enhanced survival/proliferation while improving plaque stability by augmenting cholesterol efflux and collagen deposition [82].

### 7.3. TREM2 and Microglial Regulation of Inflammation

While earlier studies characterized TREM2 primarily as an anti-inflammatory agent, recent research demonstrates its dual nature in regulating inflammation, capable of both promoting and inhibiting inflammatory responses. This binary role appears dependent on the specific pathological context and TREM2 expression levels. Studies of the senescence-accelerated mouse prone 8 (SAMP8) model have demonstrated increased TREM2 protein levels during aging. *Trem2* knockdown in SAMP8 mouse brains leads to significant increases in pro-inflammatory cytokines, including TNF-α and IL-6, while decreasing IL-10 levels [83]. Research utilizing postoperative cognitive dysfunction (POCD) mouse models and in vitro systems has indicated that TREM2 activation mitigates neuroinflammation through modulation of the PI3K/protein kinase B (Akt) signaling pathway, thereby improving postoperative learning and memory deficits [84]. In PD, TREM2 delivers substantial neuroprotection through regulation of microglial phenotypes. TREM2 knockdown enhances NLRP3 inflammasome activation and subsequent inflammatory responses, intensifying pyroptosis and dopaminergic neuron loss. Additionally, TREM2 demonstrates anti-inflammatory effects in PD through the TLR4/MyD88/NF-κB pathway [85].

Network analysis of TREM2-related gene expression in the human brain has revealed distinct clusters of both anti- and pro-inflammatory genes [86]. In patients with polycystic lipomembranous osteodysplasia with sclerosing leukoencephalopathy (PLOSL), microarray analysis of peripheral blood mononuclear cells (PBMCs) has demonstrated an upregulation of inflammation-related genes and a downregulation of innate immune response genes relative to healthy controls. Under non-disease conditions, the transcriptional profiles of bone marrow cells from *Trem2*-deficient [79] or *Trem2*-overexpressing [87] mice remain largely similar to controls [13]. However, in disease contexts, TREM2 significantly influences inflammation-related pathways. In a spinal nerve transection model, pharmacological activation of TREM2 led to elevated TNFα and IL1β levels. Notably, intrathecal administration of a TREM2 agonistic antibody in mice without nerve injuries induced pro-inflammatory cytokine expression and neuropathic pain [88]. Studies have shown [45] that soluble TREM2 (sTREM2) promotes microglial survival through PI3K/Akt-dependent mechanisms and stimulates inflammatory cytokine production. Variants of sTREM2 associated with AD risk mutations demonstrate reduced effectiveness in inhibiting apoptosis and triggering inflammatory responses. When administered to the hippocampus of wild-type and *Trem2* knockout mice, sTREM2 increases inflammatory cytokine expression and induces microglial morphological changes. These findings indicate that sTREM2 activates microglia, induces inflammatory responses, and promotes survival. Although TREM2 signaling typically exhibits anti-inflammatory effects in myeloid cells, including microglia, evidence suggests that sTREM2 may function independently of full-length TREM2 and potentially exert opposing effects on inflammation. Research has indicated that acute inflammation may initially suppress TREM2 expression, while chronic inflammation, as observed in conditions such as AD, increases TREM2 expression [89].

### 7.4. Impact of TREM2 on Microglial Phagocytosis

Recent studies have demonstrated TREM2’s crucial role in regulating microglial phagocytic capacity. TREM2 enhances microglial recognition and clearance of pathological substances through various ligand interactions. For instance, TREM2 recognizes phosphatidylserine, a signal typically exposed on apoptotic cell surfaces, initiating microglial phagocytosis [90]. TREM2 activation significantly enhances microglial phagocytic ability for Aβ plaques and synapses, thereby reducing AD-related neuronal damage [91]. In regard to PD, characterized by dopaminergic neuron loss, abnormal α-synuclein (α-syn) accumulation, and microglial activation, Yin et al. found that *Trem2* knockout mice exhibited increased pathological α-syn spread, reduced microglial reactivity, and enhanced loss of TH-positive neurons compared to wild-type mice. TREM2 overexpression enhanced reactive microglial clustering at pathological sites, suggesting TREM2 signaling maintains microglial phagocytosis, proliferation, and reactivity in PD [92]. Xie et al. discovered that TREM2 interacts with TAR-DNA-binding protein 43 kDa (TDP-43) and mediates microglial neuroprotection against TDP-43-related neurodegeneration. TREM2 deficiency impairs pathological TDP-43 clearance by microglia and enhances neuronal damage and motor deficits. Mass cytometry analysis has revealed that human TDP-43 (hTDP-43) induces a TREM2-dependent microglial subpopulation with high CD11c expression and phagocytic capacity [93]. Pang et al. demonstrated that chronic hypoperfusion induced white matter demyelination, microglial phagocytosis, and autophagy–lysosomal pathway activation, accompanied by increased TREM2 expression. TREM2 knockout attenuated white matter lesions and microglial responses, inhibited microglial phagocytosis, reduced autophagy–lysosomal pathway activation, and shifted microglial polarization toward an anti-inflammatory and homeostatic phenotype [94]. Xue et al. found that sphingosine-1-phosphate (S1P) or FTY720 (an S1P analog) promotes microglial phagocytosis in stroke. Their study confirmed that S1P has a pro-phagocytic effect on TREM2-DAP12-transfected CHO cells and *TREM2* knockdown microglia [95]. In a multiple sclerosis model, Cignarella et al. demonstrated that microglial TREM2 activation promotes myelin debris clearance and regeneration [96].

## 8. TREM2 in AD

AD is the most common neurodegenerative disorder. It is primarily characterized by a progressive decline in cognitive and memory functions. As the disease advances, patients exhibit clinical symptoms including memory loss, cognitive impairment, behavioral abnormalities, and social difficulties. The two principal pathological hallmarks of AD are extracellular amyloid plaques composed of Aβ peptides and intraneuronal neurofibrillary tangles (NFTs) formed from abnormally aggregated and hyperphosphorylated tau protein. Despite extensive research spanning over a century, the precise etiology of AD remains incompletely understood, and current therapeutic approaches are limited to symptom management, with no effective interventions capable of halting or reversing the progression of the disease.

Recent progress in whole-genome sequencing and GWASs has led to the identification of multiple genetic variants that increase the risk of developing AD. The most prevalent and extensively studied *TREM2* variant associated with an elevated the risk of developing AD is rs75932628. This single-nucleotide polymorphism (SNP) encodes a missense substitution of arginine to histidine at amino acid 47 (R47H) [89]. Jonsson et al. identified this novel risk variant, rs75932628-T, in the context of AD. Although this variant occurs less frequently than the *APOE ε4* allele, it confers a comparable risk of AD development. Since TREM2 facilitates microglial phagocytosis of amyloid plaques, reduced TREM2 activity due to the R47H substitution may result in brain damage through impaired clearance of these toxic products. Furthermore, 46 *TREM2* mutations associated with AD have been identified, including *R62H* (rs143332484), *D87N* (rs142232675), *T96K* (rs2234353), *L211P* (rs2234256), *R136Q* (rs149622783), and *p.H157Y* (rs2234255) [55,97,98,99,100,101,102], all of which increase the risk of AD development [103]. Most investigations have examined TREM2 variant effects on AD in vitro, necessitating additional animal studies to elucidate their precise pathogenic mechanisms.

In P301S tau models and senescence-accelerated SAMP8 models, transient knockdown of TREM2 increases inflammatory cytokine production [83,104]. In 5xFAD mice, TREM2 knockdown significantly elevates levels of pro-inflammatory cytokines such as IL-1β and TNF-α, exacerbating cognitive deficits [105]. Conversely, TREM2 overexpression in primary mouse glial cells and P301S mice substantially reduces inflammatory factor expression and improves neuroinflammatory responses [83,104,106]. Additionally, lipopolysaccharide (LPS) stimulation of cultured *TREM2* KO microglia results in a significant increase in inflammatory gene expression. In vitro studies have demonstrated that TREM2 overexpression can attenuate LPS-induced inflammatory responses and induce microglial polarization towards an M2 phenotype. These findings indicate that TREM2 functions in regulating both microglial inflammatory responses and phenotype modulation [105]. Some studies have also reported on TREM2’s role in promoting pro-inflammatory signaling [88,107]. Analysis of genes most strongly associated with TREM2 revealed clusters of both anti-inflammatory and pro-inflammatory genes in the brain [86]. These recent findings indicate that TREM2’s role in inflammatory processes is more complex than initially understood.

Microglia are essential immune cells in the brain that facilitate phagocytosis of Aβ plaques, and TREM2 is highly expressed in these cells. However, studies examining the impact of TREM2 deficiency on Aβ accumulation have yielded inconsistent results. Jay et al. observed that in the APPPS1-21 mouse model of AD, TREM2 deficiency improved amyloid pathology during early disease stages but worsened it in later stages [108]. In the 5xFAD mouse model, Wang et al. [79] demonstrated that TREM2 deficiency and haploinsufficiency increase Aβ accumulation due to microglial dysfunction, characterized by impaired clustering around Aβ plaques and increased apoptotic tendency. Early studies on 4-month-old 5xFAD mice revealed similar levels of microgliosis and Aβ deposition between *Trem2*-deficient and *Trem2*-sufficient mice [109]. However, TREM2 deficiency affected microglial clustering and Aβ plaque structure at early time points. *Trem2*-deficient 5xFAD mice exhibited more diffuse Aβ plaques with altered Aβ sub-species compositions, associated with increased neuroinflammation and dystrophy. Meilandt et al. [110] compared PS2APP;*Trem2* KO mice with PS2APP transgenic mice at various ages, finding increased Aβ plaque abundance at 6–7 months and a significant reduction at 12 months or 19–22 months in *Trem2*-deficient mice. These mice showed more diffuse Aβ plaques with elevated Aβ42:Aβ40 ratios and higher soluble and fibrillar Aβ oligomer levels. These studies suggest TREM2’s impact on Aβ plaque load varies with the model, age, and brain region analyzed [13,111]. Thus, TREM2 plays a dynamic and complex role in AD through its influence on microglial responses to Aβ and regulation of Aβ plaque aggregation and morphology.

In pure-tauopathy mouse models, the relationship between TREM2 and tau pathology is complex. Research has indicated that partial or normal TREM2 function can contribute to tau pathology and tau-mediated damage, while complete loss of function may reduce tau-mediated brain injuries. Leyns et al. observed no differences in tau phosphorylation and insolubility in PS19 mice with or without TREM2. Their findings suggested that in pure tauopathy, impaired microglial TREM2 signaling can decrease neuroinflammation and prevent neurodegeneration [112]. Similarly, Gratuze et al. investigated [113] the effect of the AD-associated *TREM2* variant (*R47H*) on tau-mediated neuropathology in the PS19 tauopathy mouse model. In their evaluation of PS19 mice expressing human TREM2^CV^ (the common variant) or human TREM2^R47H^, PS19-TREM2^R47H^ mice exhibited significantly reduced brain atrophy and synapse loss compared to PS19-TREM2^CV^ mice. Gene expression analysis and CD68 immunostaining indicated reduced microglial reactivity in PS19-TREM2^R47H^ mice. Furthermore, microglia expressing TREM2^R47H^ in PS19 mouse and human AD brains exhibited reduced phagocytosis of postsynaptic elements. These findings suggest that impaired TREM2 signaling reduces microglia-mediated neurodegeneration in tauopathy. In contrast, Lee et al. examined [114] *Trem2* deficiency effects in pR5-183 and TauPS2APP mouse models, yielding different results. In these models, Aβ pathology intensified tau pathology and neurodegeneration. Single-cell RNA sequencing revealed distinct, Aβ- and TREM2-dependent activation of DAM in TauPS2APP mice. With Aβ pathology present, TREM2 deficiency further increased tau accumulation and spread and accelerated brain atrophy. Without Aβ pathology, TREM2 deficiency had no significant effect on these pathological processes. Thus, TREM2 appears to play a dynamic and complex role in AD progression, potentially modulating tau-driven neurodegeneration by limiting Aβ-promoted pathogenic tau propagation. This TREM2-dependent microglial function proves particularly crucial in preventing tau accumulation and spread, especially in the context of Aβ pathology.

TREM2 maintains a close association with lipid metabolism in microglia [79,115]. TREM2 binds to lipid-related ligands, including phospholipids, high-density lipoprotein (HDL), LDL, and APOE [116,117,118]. APOE, the primary cholesterol transporter in the CNS, is expressed in both microglia and astrocytes. TREM2 regulates APOE expression on microglia, influencing lipid uptake and metabolism, which may explain cholesterol ester accumulation in TREM2-deficient microglia. TREM2 function loss reduces plaque-associated APOE [111,119]. Moreover, *Trem2* deficiency exacerbates neurodegeneration in tau mutant mice expressing human APOE4 [14].

Changes in the levels of AD-related proteins, such as Aβ42 and tau, in CSF reflect the presence and progression of AD in the brain. A reduction in CSF Aβ42 levels represents one of the earliest detectable fluid biomarkers of AD pathology. Although low CSF Aβ42 levels predict future cognitive impairment, individuals with reduced CSF Aβ42 levels may remain asymptomatic for years [120]. Following the identification of TREM2 variants associated with AD, several studies have demonstrated that CSF sTREM2 level changes may indicate inflammatory processes associated with the transition from preclinical to clinical AD [20,121]. Studies on late-onset AD and autosomal dominant AD populations have revealed elevated sTREM2 levels in the CSF of AD patients, showing positive correlation with tau and p-tau levels. However, researchers observed no significant correlation between sTREM2 and Aβ42 levels [10,11,122,123].

## 9. TREM2 in PD

PD is the second most common neurodegenerative disorder after AD [124]. Research has demonstrated that immune pathway dysregulation, including alterations in cytokine signaling, immune cell proliferation and migration, and phagocytosis, is closely linked to dopaminergic (DA) neurodegeneration [125]. During DA neurodegeneration, microglial activation increases alongside inflammatory mediator accumulation [126]. Consequently, microglia-mediated neuroinflammation plays a crucial role in the pathogenesis of PD [127]. The aggregation of α-synuclein (α-syn) monomers into amyloid fibrils through oligomeric intermediates is a toxic mechanism leading to PD. Yin et al. demonstrated that microglia phagocytose pre-formed α-syn fibrils in a concentration- and time-dependent manner, showing a capacity to degrade α-syn aggregates. In PD studies, *Trem2* knockout mice displayed enhanced pathological α-syn spread, decreased microglial reactivity, and increased loss of TH-positive neurons compared with wild-type mice, indicating that TREM2 modulation may benefit PD treatment [92]. Similarly, Guo et al. utilized a PD model to demonstrate that TREM2 deficiency both in vitro and in vivo triggered a shift from anti-inflammatory to pro-inflammatory microglial activation states, compromising TREM2 signaling and intensifying the pro-inflammatory response to α-syn, thus aggravating α-syn-induced neurodegeneration [128]. Multiple studies have identified TREM2 as a significant risk factor for PD, suggesting its potential as a therapeutic target for PD and other neurodegenerative diseases. The *TREM2* variant rs75932628 (*p.R47H*) has been associated with increased PD risk [53,129]. However, studies from Greece and China indicate that *R47H* may not represent a major genetic risk factor for PD [130,131]. A case–control study involving a Chinese population revealed significantly elevated sTREM2 levels in the CSF of PD patients compared to that in healthy controls, though no significant plasma upregulation was observed [132]. Furthermore, Zhang et al. found higher CSF sTREM2 levels in late-onset versus early-onset PD patients, with baseline sTREM2 levels predicting motor progression over a 4-year follow-up [133]. Essential tremor (ET) represents the most common movement disorder in adults, with an unclear pathophysiology [134]. Evidence suggests ET shares common etiologies with other neurodegenerative diseases such as AD and PD [85,135]. A cross-sectional, multicenter international study identified a significant association between the *TREM2 p.R47H* variant and ET in a Spanish cohort, though this association was not replicated in other studied populations.

## 10. TREM2 in ALS

ALS is a fatal neurodegenerative disease characterized by progressive, painless muscle weakness resulting from motor neuron degeneration in the brain and spinal cord. The condition manifests clinically as progressive loss of motor function, ultimately leading to death, typically via respiratory failure [136]. Our current understanding indicates that ALS not only affects motor neurons but also involves microglial activation and enhanced inflammatory responses from peripheral lymphocytes and macrophages, which intensify disease progression [137]. TDP-43, essential for gene expression regulation, constitutes a primary component of insoluble and ubiquitinated protein aggregates present in most ALS patients [138]. Research utilizing viral-mediated and transgenic mouse models has examined microglial TREM2’s role in TDP-43-related neurodegeneration. These investigations have revealed that TREM2 deficiency compromises the capacity of microglia to phagocytose and eliminate pathological TDP-43, resulting in increased neuronal damage and motor dysfunction. Additional studies have demonstrated that microglial TREM2 interacts with pathological human TDP-43 (hTDP-43), suggesting a molecular mechanism through which microglia provide neuroprotection in TDP-43-mediated neurodegeneration [93]. A case–control study demonstrated elevated overall and transcript-specific *TREM2* mRNA levels in ALS patients’ spinal cords compared to controls, with corresponding changes in TREM2 protein levels. Furthermore, sTREM2 levels in both CSF and serum were markedly elevated in ALS patients versus controls, suggesting potential non-invasive biomarkers for ALS [42]. The GGGGCC hexanucleotide repeat expansion in the C9orf72 gene represents the most prevalent genetic factor in ALS and FTD. Research has revealed interaction between NLRP3 and TREM2 signaling, indicating that modulating the NLRP3 inflammasome to maintain TREM2 function could offer therapeutic potential for C9orf72-ALS/FTD [139]. Janet Cady et al. identified the *TREM2 p.R47H* variant as a significant risk factor for sporadic ALS [54]. However, Vasileios Siokas et al. found no significant association between the *TREM2* rs75932628-T variant and ALS, suggesting limited involvement in ALS pathophysiology [140]. Similarly, a large-scale Chinese study detected no *TREM2* rs75932628-T variant in sporadic ALS patients or controls, indicating a minimal contribution to ALS pathogenesis in this population [141]. Data-driven analysis has indicated that CSF soluble TREM2 protein levels may predict survival in late-stage ALS patients, suggesting TREM2’s potential as a therapeutic target [142]. While genetic evidence connects TREM2 to ALS, its precise role in pathogenesis and therapeutic development requires further investigation.

## 11. TREM2 in NHD

NHD, also known as polycystic lipomembranous osteodysplasia with sclerosing leukoencephalopathy (PLOSL; OMIM 221770, OMIM 605086), is a rare autosomal recessive disorder characterized by rapid psychiatric symptom development progressing to presenile dementia and bone cysts confined to the wrists and ankles. NHD results from homozygous pathogenic mutations in *TREM2* or TYRO protein tyrosine kinase-binding protein (TYROBP, also known as DAP12 genes) [143]. Established mutations in *TREM2*’s extracellular domain associated with NHD include *Y38C*, *W50C*, *T66M* and *V126G* [58,60,144,145]. Fabia Filipello et al. demonstrated that the *TREM2 p.Q33X* mutation in microglia causes lysosomal dysfunction, while compounds targeting lysosomal biogenesis can ameliorate numerous NHD microglial defects. Efthimios Dardiotis et al. [50] documented a 33-year-old Greek woman presenting with an NHD-suggestive phenotype. Whole-genome sequencing identified a novel mutation in *TREM2* gene exon 2, *c.244G>T* (*p.W50C*), in a heterozygous state in the patient and family members, expanding the spectrum of TREM2 mutations associated with the NHD phenotype [145]. Additionally, Y. Numasawa described a Japanese NHD family affected by a *TREM2* splicing mutation. A homozygous T-to-C conversion at intron 3’s second position (*c.482+2T>C*) in the splice donor consensus site was identified, causing exon 3 skipping and truncated protein expression. This mutation triggers neuroinflammation and neurodegeneration [146].

## 12. TREM2 in Other Neurological Disorders

### 12.1. Multiple Sclerosis (MS)

MS is a CNS demyelinating disorder characterized by combined inflammatory and neurodegenerative mechanisms. While current immunomodulatory therapies address neuroinflammation, effective strategies for myelin regeneration and disease progression prevention have not yet been established [147]. Microglia play a crucial role in clearing myelin debris from demyelinated areas, an essential step enabling remyelination [148]. Brain pathology observed in NHD patients indicates that disruption of the TREM-2/DAP12 pathway leads to neurodegeneration accompanied by demyelination and axonal loss [49]. Laura Piccio et al. demonstrated that TREM2 expression increases in the spinal cord during both early inflammatory and chronic phases of experimental autoimmune encephalomyelitis (EAE) induced by myelin oligodendrocyte glycoprotein (MOG) peptide. Their research showed that TREM2 exhibits high expression on microglia in the CNS during EAE, and TREM2 blockade during the effector phase of EAE exacerbates disease progression, diffuse CNS inflammatory infiltration, and demyelination, indicating TREM2’s critical role in CNS inflammatory response [149]. Cignarella et al. identified that TREM2 shows high expression on microglia/macrophages in active MS lesions. In cuprizone (CPZ)-induced demyelination, TREM2 deficiency impaired myelin clearance, while in vivo treatment with AL002a enhanced efficient myelin debris clearance, improving myelin phagocytosis and intracellular degradation after CPZ-induced demyelination [96]. MS is mediated by pathogenic helper T cell 17 (Th17) cells. Research has indicated that TREM2 exhibits high expression on pathogenic CD4-positive T lymphocytes (CD4 T cells) in MS patients and in the EAE mouse model. Further mechanistic studies have revealed that the TREM-2/ZAP70 signal transducer and activator of transcription 3 (STAT3) signaling axis is essential for the activation and differentiation of Th17 cells during EAE progression [150].

### 12.2. Neuromyelitis Optica Spectrum Disorder (NMOSD)

NMOSD is an inflammatory demyelinating disease of the CNS triggered by autoimmune mechanisms. Microglia become activated and play a critical role in responding to tissue damage. In an NMOSD mouse model induced by Aquaporin-4 (AQP4)-IgG and complement, TREM2 deficiency intensified brain injury, decreased microglial clearance of myelin debris, and impaired the differentiation of oligodendrocyte precursor cells (OPCs), ultimately resulting in failed remyelination [151]. Chuan Qin et al. employed Mendelian randomization analysis to demonstrate that elevated CSF sTREM2 levels are genetically responsible for the risk of AQP4-IgG seropositive NMOSD. Increased CSF sTREM2 levels correlated positively with microglia/macrophage activation, neuroinflammation, and subsequent myelin damage in patients and NMOSD mouse models. Additional mechanistic studies revealed that sTREM2 exerts pathological effects on microglia/macrophages through the HSP70-NF-κB signaling pathway [152].

### 12.3. Cerebral Small-Vessel Disease (SVD)

Cerebral SVD constitutes a major cause of stroke and vascular cognitive impairment, characterized by lesions in the small and deep penetrating arteries and arterioles of the brain [153]. Cerebral amyloid angiopathy (CAA) and hypertensive SVD (SVD-HTN) represent the most common sporadic forms of SVD. Preclinical evidence emphasizes the significant contribution of neuroinflammation to CAA progression, with activated microglia specifically clustering around CAA vessels with total amyloid deposits [154]. Targeting inflammation emerges as a novel therapeutic approach for SVD. In a cross-sectional study comparing plasma soluble TREM2 levels in 10 AD patients and 66 survivors of spontaneous intracerebral hemorrhage with cerebral amyloid angiopathy or hypertensive small-vessel disease, plasma-soluble TREM2 demonstrated an association with white matter hyperintensities, independently of amyloid and tau pathology. These findings highlight plasma soluble TREM2’s potential utility as a predictive marker for white matter injury in SVD and the clinical significance of targeting innate immune responses in treatment [153]. In a bilateral carotid artery stenosis (BCAS) mouse model of chronic cerebral hypoperfusion, chronic hypoperfusion-induced white matter demyelination, microglial phagocytosis, and activation of the microglial autophagy–lysosomal pathway, accompanied by an increase in TREM2 expression. TREM2 knockout resulted in attenuated white matter lesions and microglial responses [94]. In a traumatic brain injury (TBI) mouse model, TREM2 expression peaked 3 days post-TBI, primarily localized to microglia within white matter. TREM2 depletion worsened abnormal neurobehavioral outcomes, while TREM2 upregulation mitigated white matter injury, promoted oligodendrocyte regeneration, and facilitated neurobehavioral recovery after TBI [155].

## 13. Therapeutic Potential of TREM2 in Future Applications

TREM2 holds significant promise as a therapeutic target for neurodegenerative diseases, demonstrating considerable potential in drug development and treatment strategies. The recent progress of TREM2-targeted therapies is presented in Table 2.

The first agonistic antibody targeting TREM2, the monoclonal antibody 4D9, demonstrates reduced amyloid plaque burden in AD mouse models [156]. The epitope of 4D9 localizes to the stalk region of TREM2 near the cleavage site, enabling a dual mechanism of action: stabilization of TREM2 on the cell surface to reduce shedding and activation of downstream phosphorylated SYK signaling. In mouse models of AD-related pathology, 4D9 attenuated amyloid deposition, increased TREM2 expression in microglia, and reduced levels of a homeostatic marker, indicating a protective role through driving microglia toward a disease-associated state.

AL002 is an investigational, engineered, humanized monoclonal immunoglobulin G1 (IgG1) antibody designed to target TREM2. Studies on its variant, AL002c, revealed that acute systemic administration of AL002c induced microglial proliferation in both CV and *R47H* transgenic mice, as demonstrated by single-cell RNA sequencing. Chronic administration of AL002c reduced filamentous plaques and neuritic dystrophy, modulated behavioral outcomes, and attenuated microglial inflammatory responses [157]. In a preclinical and first-in-human Phase II, randomized, placebo-controlled, double-blind study, researchers evaluated the safety, tolerability, pharmacokinetics, and pharmacodynamics of AL002 in healthy volunteers following single ascending dose (SAD) administration. The findings support the continued clinical development of AL002 for AD and other neurodegenerative disorders [158]. Currently, a Phase II, randomized, double-blind, placebo-controlled study targeting early-stage AD is underway. Another promising antibody, DNL919 (ATV:TREM2), is a high-affinity human TREM2-activating antibody featuring a monovalent transferrin receptor (TfR) binding site, termed an antibody transport vehicle (ATV), which facilitates transcytosis across the blood–brain barrier. Compared with standard anti-TREM2 antibodies, ATV:TREM2 demonstrated improved brain biodistribution and enhanced signaling following peripheral delivery in mice. In human iPSC-derived microglia, ATV:TREM2 induced proliferation and improved mitochondrial metabolism. Single-cell RNA sequencing and morphometric analyses revealed that ATV:TREM2 shifted microglia into a metabolically responsive state distinct from that induced by amyloid pathology. In AD mouse models, ATV:TREM2 enhanced cerebral microglial activity and glucose metabolism [12]. Michael Fassler and colleagues developed a novel monoclonal antibody targeting both membrane-bound and soluble TREM2. The selected antibody promoted microglial proliferation, uptake of oligomeric β-amyloid/apoptotic neurons, and activation in a SYK- and AKT-dependent manner through binding to membrane-bound TREM2. The antibody also demonstrated strong binding to soluble TREM2 in the cerebrospinal fluid of AD patients and attenuated pro-inflammatory programs triggered by intracerebral antibody injection. In vivo therapeutic administration improved cognitive function in experimental amyloidosis models and enhanced plaque-associated microglial coverage and activation [159].

Recent evidence indicates that certain natural compounds exhibit therapeutic potential for neurodegenerative diseases such as AD by modulating TREM2-related molecular pathways. Curcumin exhibits anti-inflammatory and neuroprotective properties. In animal models of cognitive impairment induced by diabetes mellitus and chronic cerebral hypoperfusion (DM/CCH), curcumin exhibited protective effects by inhibiting microglial activation-induced neuroinflammation, modulating the TREM2/TLR4/NF-κB pathway, attenuating apoptosis, and reducing NLRP3-dependent pyroptosis [160]. In APPsw transgenic mice, curcumin enhanced amyloid phagocytic clearance by downregulating CD33 and upregulating TREM2 and TyroBP while restoring neuroinflammatory networks associated with neurodegeneration [161]. Echinacoside (EDB) alleviates hippocampal inflammatory responses in APP/PS1 mice, an effect linked to the TREM2/TLR4/MAPK signaling pathway. Additional in vitro studies revealed that EDB suppresses neuroinflammation in LPS-stimulated BV2 cells by inhibiting the TLR4/MAPK signaling pathway and upregulating TREM2 expression [162]. Wang et al. demonstrated through in vivo and in vitro experiments that Yishen Huazhuo decoction modulates microglial polarization and reduces AD-associated neuroinflammation by regulating the TREM2/NF-κB signaling pathway [163]. Similarly, Hydroxysafflor Yellow A (HSYA) regulates microglial inflammatory phenotypes by modulating Aβ1-42-induced polarization of BV2 microglia (M1/M2), a process potentially mediated through the TREM2/TLR4/NF-κB pathway [164].

**Figure 3:**
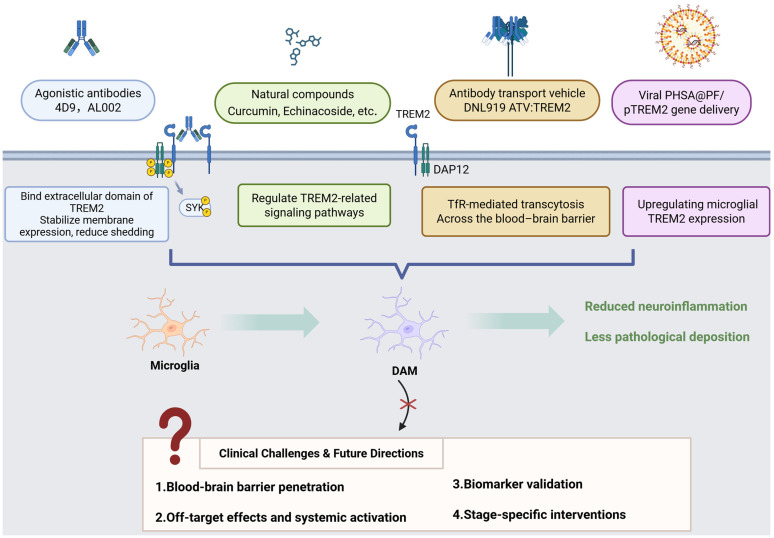
Therapeutic strategies targeting TREM2. This figure summarizes key therapeutic strategies to enhance TREM2 function in microglia, including agonistic antibodies (e.g., 4D9), natural compounds, antibody transport vehicles (e.g., DNL919), and viral gene delivery systems. These approaches aim to stabilize TREM2 signaling, reduce neuroinflammation, and limit pathological protein deposition. Major clinical challenges such as BBB penetration, off-target effects, biomarker development, and stage-specific treatment are also highlighted. The images were created using licensed Biorender online software (https://app.biorender.com/).

Clinically, TREM2-targeted therapies are hindered by significant challenges, including limited blood–brain barrier penetration and unintended systemic effects (such as increased atherosclerosis). Although CSF-soluble TREM2 (sTREM2) demonstrates correlations with tau burden in AD, its variable expression in PD and ALS, combined with confounding factors like the H157Y mutation that increases sTREM2 shedding, highlights the necessity of comprehensive longitudinal biomarker validation. Future research priorities include utilizing single-cell omics to characterize TREM2-dependent microglial subpopulations (particularly disease-associated microglia), implementing conditional modulation models to examine temporal relationships in Aβ/tau dynamics, and integrating multi-omics approaches to identify novel therapeutic targets (especially in regard to lipid metabolism). Successfully addressing these complexities requires careful consideration of pathway interactions, stage-specific biological processes, and translational viability to effectively utilize TREM2’s dual functions. Figure 3 provides an overview of key therapeutic approaches for enhancing TREM2 function in microglia while also addressing major clinical challenges currently encountered.

**Table 2:** Research progress regarding TREM2-targeted therapies.

Drug Name	Drug Type	Mechanism of Action	Indication	Development Stage	Notes	References	Registration Number
4D9	Monoclonal antibody (agonist)	Stabilizes TREM2 on the cell surface and inhibits shedding; activates TREM2-DAP12/SYK signaling through phospho-SYK.	AD	_	Reduces amyloidogenesis and homeostatic markers. Enhances microglial TREM2 expression in AD models.	[156,165]	_
AL002	Humanized mAb (agonist)	Activates TREM2 signaling.	AD	Phase II completed (2024)	Enhances microglial activity to reduce Aβ plaques and neuroinflammation.	[157,158]	NCT04592874
Iluzanebart (VGL101)	Humanized mAb (agonist)	Enhances the TREM2-DAP12/SYK pathway.	AD, adult-onset leukoencephalopathy (ALSP)	Phase II	Well-tolerated; monthly dosing reduces CSF sTREM2 levels.	[166]	NCT05677659
PY314	Humanized mAb (antagonist)	Blocks TREM2 and depletes tumor-associated macrophages.	Solid tumors (e.g., ovarian cancer)	Phase Ib	Combined with PD-1 inhibitors for platinum-resistant ovarian cancer.	[167,168]	NCT04691375
VHB937	Humanized mAb (agonist)	Activates TREM2 and reduces shedding.	Neurodegeneration, metabolic syndrome	Preclinical/Phase I (2024 filing)	Enhances microglial phagocytosis; neuroprotective in animal models.	[169]	NCT06643481
DNL919 (TAK-920)	Monoclonal antibody (agonist)	Activates TREM2 signaling.	AD	Discontinued (2023)	Phase I showed hematologic toxicity; narrow therapeutic window.	[12]	NCT05450549
Ab18 TVD-Ig/αTfR	Tetravalent bispecific antibody	Enhances TREM2 clustering and blood–brain barrier (BBB) penetration.	AD	Preclinical	Engineered antibody reduces Aβ burden and improves cognition in mice.	[170]	_

## 14. Conclusions

This review analyzed the structure and function of TREM2 and its influence on microglia, synthesizing our current understanding of its role in neurodegenerative diseases, particularly AD, with an additional focus on PD and ALS. The field has undergone rapid advancement through mechanistic studies and therapeutic developments, notably with monoclonal antibodies progressing to Phase II clinical trials for AD. A significant challenge remains: TREM2-mediated immune stimulation lacks cellular specificity, potentially resulting in off-target effects that may impact therapeutic efficacy and safety. Future research must address several critical aspects: determining the context-dependent approach of activating versus inhibiting TREM2, establishing the optimal therapeutic window, and further elucidating signaling pathways to develop precise immune modulation strategies. Addressing these challenges is essential for effectively exploiting TREM2’s therapeutic potential for neurodegenerative diseases.

## Data Availability

Not applicable.

## Abbreviations

The following abbreviations are used in this manuscript:

AD	Alzheimer’s Disease
PD	Parkinson’s Disease
ALS	Amyotrophic Lateral Sclerosis
TREM2	Triggering Receptor Expressed on Myeloid Cells 2
GWASs	Genome-Wide Association Studies
DAM	Disease-Associated Microglia
ITAM	Immunoreceptor Tyrosine-based Activation Motif
DAP12	DNAX-Activating Protein 12
SYK	Spleen Tyrosine Kinase
PI3K	Phosphatidylinositol 3-Kinase
PLCγ	Phospholipase C gamma
IP3	Inositol Trisphosphate
DAG	Diacylglycerol
mTOR	Mechanistic Target of Rapamycin
MAPK	Mitogen-Activated Protein Kinase
TLR4	Toll-Like Receptor 4
NF-κB	Nuclear Factor kappa B
NLRP3	NOD-Like Receptor family Pyrin domain containing 3
IL-1β	Interleukin-1 beta
TNF-α	Tumor Necrosis Factor alpha
IL-6	Interleukin-6
IL-8	Interleukin-8
NHD	Nasu–Hakola Disease
FTD	Frontotemporal Dementia
sTREM2	Soluble TREM2
CSF	Cerebrospinal Fluid
SNP	Single-Nucleotide Polymorphism
ADAM10/17	A Disintegrin and Metalloproteinase 10/17
BBB	Blood–Brain Barrier
PET	Positron Emission Tomography
APOE	Apolipoprotein E
Aβ	Amyloid-beta
NFTs	Neurofibrillary Tangles
LPS	Lipopolysaccharide
MOG	Myelin Oligodendrocyte Glycoprotein
EAE	Experimental Autoimmune Encephalomyelitis
MS	Multiple Sclerosis
NMOSD	Neuromyelitis Optica Spectrum Disorder
SVD	Small Vessel Disease
CAA	Cerebral Amyloid Angiopathy
BCAS	Bilateral Carotid Artery Stenosis
CD33	Cluster of Differentiation 33
TDP-43	TAR DNA-binding protein 43
CNS	Central Nervous System
HDL	High-Density Lipoprotein
LDL	Low-Density Lipoprotein
TfR	Transferrin Receptor
TBI	Traumatic Brain Injury
Th17	T helper 17 cells
ZAP70	Zeta-chain-Associated Protein Kinase 70
STAT3	Signal Transducer and Activator of Transcription 3
AQP4	Aquaporin-4
OPCs	Oligodendrocyte Precursor Cells
SVD-HTN	Hypertensive Small Vessel Disease
ALSP	Adult-Onset Leukoencephalopathy
TREM	Triggering Receptor Expressed on Myeloid Cells
TYROBP	TYRO Protein Tyrosine Kinase-Binding Protein
PLOSL	Polycystic Lipomembranous Osteodysplasia with Sclerosing Leukoencephalopathy
BV2	Murine Microglial Cell Line
CHO	Chinese Hamster Ovary
iPSC	Induced Pluripotent Stem Cell
BMDMs	Bone Marrow-Derived Macrophages
CPZ	Cuprizone
LXR	Liver X Receptor
ACAT1	Acyl-CoA Cholesterol Acyltransferase 1
oxLDL	Oxidized Low-Density Lipoprotein
ABCA1	ATP-Binding Cassette Sub-family A Member 1
SHIP1	Src Homology 2-containing Inositol Phosphatase 1
SHIP2	Src Homology 2-containing Inositol Phosphatase 2
S1P	Sphingosine-1-phosphate
TREM2R47H	TREM2 R47H Variant
TREM2CV	TREM2 Common Variant
TREM2KO	TREM2 Knockout
TREM2−/−	TREM2-Deficient
SAMP8	Senescence-Accelerated Mouse Prone 8
POCD	Postoperative Cognitive Dysfunction
MPTP	1-Methyl-4-phenyl-1,2,3,6-tetrahydropyridine
α-syn	α-Synuclein
TH	Tyrosine Hydroxylase
hTDP-43	Human TAR DNA-binding Protein 43
hTREM2	Human TREM2
CD11c	Cluster of Differentiation 11c
CD68	Cluster of Differentiation 68
CD4	Cluster of Differentiation 4
MHC-I	Major Histocompatibility Complex Class I
MHC-II	Major Histocompatibility Complex Class II
LAM	Lipid-Associated Macrophages
ATP	Adenosine Triphosphate
HSP70	Heat Shock Protein 70
GLUT5	Glucose Transporter 5
SPP1	Secreted Phosphoprotein 1
p-SYK	Phosphorylated SYK
FDG	Fluorodeoxyglucose
